# Morpholinium styphnate

**DOI:** 10.1107/S1600536810038304

**Published:** 2010-10-02

**Authors:** D. Kalaivani, R. Malarvizhi

**Affiliations:** a PG & Research Department of Chemistry, Seethalakshmi Ramaswami College, Tiruchirappalli 620 002, Tamil Nadu, India

## Abstract

In the title mol­ecular salt (systematic name: morpholinium 3-hy­droxy-2,4,6-trinitro­phenolate), C_4_H_10_NO^+^·C_6_H_2_N_3_O_8_^−^, two of the nitro groups of the anion are close to parallel with the plane of the benzene ring [dihedral angles = 3.46 (9) and 11.60 (10)°] and one is almost perpendicular [dihedral angle = 82.23 (8)°]. An intra­molecular O—H⋯O hydrogen bond occurs in the anion. The morpholinium cation has a slightly distorted chair conformation. In the crystal, the components are linked by simple N—H⋯O and trifurcated N—H⋯(O,O,O) hydrogen bonds.

## Related literature

For related mol­ecular salts, see: Radha *et al.* (1987 ▶).
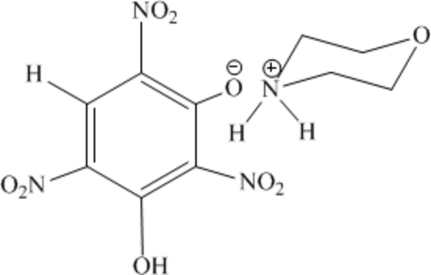


## Experimental

### 

#### Crystal data

C_4_H_10_NO^+^·C_6_H_2_N_3_O_8_^−^
*M* *_r_* = 332.24
Triclinic, 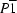

*a* = 7.680 (5) Å
*b* = 7.973 (5) Å
*c* = 11.852 (5) Å
α = 94.785 (5)°
β = 99.016 (5)°
γ = 108.188 (5)°
*V* = 674.1 (7) Å^3^
*Z* = 2
Mo *K*α radiation
μ = 0.15 mm^−1^
*T* = 293 K
0.30 × 0.16 × 0.16 mm

#### Data collection

Bruker Kappa APEXII CCD diffractometer
Absorption correction: multi-scan (*SADABS*; Bruker, 1999 ▶) *T*_min_ = 0.957, *T*_max_ = 0.977
16214 measured reflections
3841 independent reflections
2944 reflections with *I* > 2σ(*I*)
*R*_int_ = 0.023

#### Refinement

*R*[*F* ^2^ > 2σ(*F* ^2^)] = 0.050
*wR*(*F* ^2^) = 0.157
*S* = 1.06
3841 reflections
225 parameters
H atoms treated by a mixture of independent and constrained refinement
Δρ_max_ = 0.77 e Å^−3^
Δρ_min_ = −0.38 e Å^−3^

### 

Data collection: *APEX2* (Bruker, 2004 ▶); cell refinement: *SAINT-Plus* (Bruker, 2004 ▶); data reduction: *SAINT-Plus*; program(s) used to solve structure: *SIR92* (Altomare *et al.*, 1993 ▶); program(s) used to refine structure: *SHELXL97* (Sheldrick, 2008 ▶); molecular graphics: *ORTEP-3* (Farrugia, 1997 ▶) and *Mercury* (Macrae *et al.*, 2006 ▶); software used to prepare material for publication: *SHELXL97*.

## Supplementary Material

Crystal structure: contains datablocks global, I. DOI: 10.1107/S1600536810038304/hb5640sup1.cif

Structure factors: contains datablocks I. DOI: 10.1107/S1600536810038304/hb5640Isup2.hkl

Additional supplementary materials:  crystallographic information; 3D view; checkCIF report

## Figures and Tables

**Table 1:** Hydrogen-bond geometry (Å, °)

*D*—H⋯*A*	*D*—H	H⋯*A*	*D*⋯*A*	*D*—H⋯*A*
O8—H8⋯O5	0.91 (3)	1.75 (3)	2.571 (2)	149 (2)
N4—H4*A*⋯O7	0.87 (2)	1.87 (2)	2.715 (2)	164 (2)
N4—H4*B*⋯O2^i^	0.86 (2)	2.31 (2)	2.962 (2)	132.4 (17)
N4—H4*B*⋯O5^ii^	0.86 (2)	2.42 (2)	2.967 (2)	121.5 (17)
N4—H4*B*⋯O3^iii^	0.86 (2)	2.54 (2)	3.206 (3)	134.3 (17)

Symmetry codes: (i) 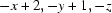; (ii) 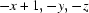; (iii) 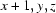.

**Fig. 1.**
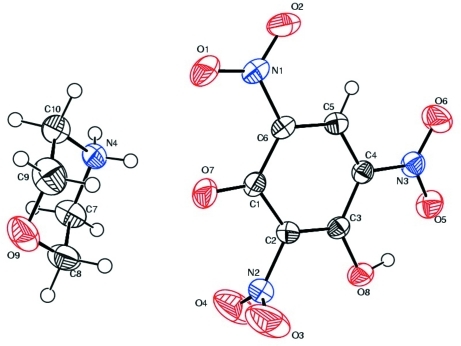
The molecular structure of (I) showing 50% displecement ellipsoids.

**Fig. 2.**
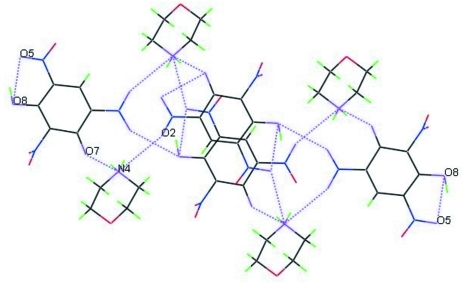
The hydrogen bonding pattern

**Fig. 3.**
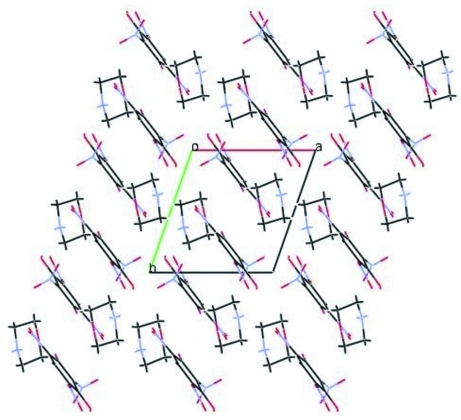
The packing view of the adduct

## supplementary crystallographic information

### Comment

Picric acid (1-hydroxy – 2,4,6- trinitrobenzene) forms 1:1 donor –acceptor adducts with amines and in these adducts, the main stabilizing factor is the proton transfer from OH of nitro compound to the nitrogen atom of amine (Radha *et al.*,1987). Unlike picric acid, styphnic acid (2,4,6-trinitro -1,3-benzene diol) contains two phenolic OH groups and hence the type of adduct formation with amines and the mode of interaction are to be envisaged. This necessitates the synthesis of the title molecule from styphnic acid and morpholine (tetrahydro – 1,4-oxazine). Single crystal X-ray analysis data clearly indicate that 1:1 adduct (Scheme 1)is formed from styphnic acid and morpholine and the main contributing factor for the formation of the adduct is the proton transfer from phenolic OH of styphnic acid to the nitrogen atom of morpholine. Hydrogen bond (N—H···O) is noticed between cation and anion moieties. Intramolecular hydrogen bond [O—H···O; *R*_1_^1^(6) motif] is also observed. Puckering parameters[(q2 = 0.0243(0.0017), q3=0.5764 (0.0019), φ2=-175.98(5.69), QT=0.5770(0.0019);θ2=2.41(0.17)] of the cation moiety (morpholinium ion) indicate that it has slightly distorted chair conformation.

### Experimental

Styphnic acid (2.45 g, 0.01 mol) was dissolved in the minimum quantity of ethanol. Morpholine(0.90 g, 0.01 mol) dissolved in the minimum amount of ethanol was added to styphnic acid solution. The mixture was stirred well for 3 h and kept as such for another 6 h. The mixture was then poured into ice cold water with stirring. The adduct formed was filtered and washed first with water and then with alcohol and dried. The dried adduct was washed several times with ether and recrystallized from ethanol (yield 70–75% mp.481–483 K). Yellow prisms of (I) were obtained by slow evaporation of ethanol at room temperature. The same product was obtained when styphnic acid (0.01 mol) was mixed with excess morpholine (0.03 mol).

### Refinement

The highset difference peak is 0.90Å from O4.

### Crystal data

**Table:**

C_4_H_10_NO^+^·C_6_H_2_N_3_O_8_^−^	*Z* = 2
*M**_r_* = 332.24	*F*(000) = 344
Triclinic, *P*1	*D*_x_ = 1.637 Mg m^−^^3^
Hall symbol: -P 1	Mo *K*α radiation, λ = 0.71073 Å
*a* = 7.680 (5) Å	Cell parameters from 5851 reflections
*b* = 7.973 (5) Å	θ = 1.8–29.8°
*c* = 11.852 (5) Å	µ = 0.15 mm^−^^1^
α = 94.785 (5)°	*T* = 293 K
β = 99.016 (5)°	Prism, yellow
γ = 108.188 (5)°	0.30 × 0.16 × 0.16 mm
*V* = 674.1 (7) Å^3^	

### Data collection

**Table:**

Bruker Kappa APEXII CCD diffractometer	3841 independent reflections
Radiation source: fine-focus sealed tube	2944 reflections with *I* > 2σ(*I*)
graphite	*R*_int_ = 0.023
ω and φ scan	θ_max_ = 29.8°, θ_min_ = 1.8°
Absorption correction: multi-scan (*SADABS*; Bruker, 1999)	*h* = −10→10
*T*_min_ = 0.957, *T*_max_ = 0.977	*k* = −11→11
16214 measured reflections	*l* = −16→16

### Refinement

**Table:**

Refinement on *F*^2^	Secondary atom site location: difference Fourier map
Least-squares matrix: full	Hydrogen site location: inferred from neighbouring sites
*R*[*F*^2^ > 2σ(*F*^2^)] = 0.050	H atoms treated by a mixture of independent and constrained refinement
*wR*(*F*^2^) = 0.157	*w* = 1/[σ^2^(*F*_o_^2^) + (0.0867*P*)^2^ + 0.1583*P*] where *P* = (*F*_o_^2^ + 2*F*_c_^2^)/3
*S* = 1.06	(Δ/σ)_max_ < 0.001
3841 reflections	Δρ_max_ = 0.77 e Å^−^^3^
225 parameters	Δρ_min_ = −0.38 e Å^−^^3^
0 restraints	Extinction correction: *SHELXL97* (Sheldrick, 1997), Fc^*^=kFc[1+0.001xFc^2^λ^3^/sin(2θ)]^-1/4^
Primary atom site location: structure-invariant direct methods	Extinction coefficient: 0.049 (7)

### Special details

**Table:**

Geometry. All e.s.d.'s (except the e.s.d. in the dihedral angle between two l.s. planes) are estimated using the full covariance matrix. The cell e.s.d.'s are taken into account individually in the estimation of e.s.d.'s in distances, angles and torsion angles; correlations between e.s.d.'s in cell parameters are only used when they are defined by crystal symmetry. An approximate (isotropic) treatment of cell e.s.d.'s is used for estimating e.s.d.'s involving l.s. planes.
Refinement. Refinement of *F*^2^ against ALL reflections. The weighted *R*-factor *wR* and goodness of fit *S* are based on *F*^2^, conventional *R*-factors *R* are based on *F*, with *F* set to zero for negative *F*^2^. The threshold expression of *F*^2^ > σ(*F*^2^) is used only for calculating *R*-factors(gt) *etc*. and is not relevant to the choice of reflections for refinement. *R*-factors based on *F*^2^ are statistically about twice as large as those based on *F*, and *R*- factors based on ALL data will be even larger.

### Fractional atomic coordinates and isotropic or equivalent isotropic displacement parameters (Å^2^)

**Table:**

	*x*	*y*	*z*	*U*_iso_*/*U*_eq_	
C1	0.48003 (18)	0.27015 (18)	−0.13460 (12)	0.0293 (3)	
C2	0.31234 (18)	0.11938 (18)	−0.14972 (11)	0.0287 (3)	
C3	0.24207 (17)	0.02930 (17)	−0.06486 (12)	0.0270 (3)	
C4	0.33875 (18)	0.09746 (17)	0.05024 (11)	0.0274 (3)	
C5	0.49948 (18)	0.24441 (18)	0.07302 (12)	0.0288 (3)	
C6	0.57105 (17)	0.32628 (17)	−0.01487 (12)	0.0278 (3)	
C7	0.7665 (3)	0.2429 (2)	−0.41273 (15)	0.0457 (4)	
H7A	0.7022	0.1253	−0.3954	0.055*	
H7B	0.8644	0.2344	−0.4533	0.055*	
C8	0.6313 (3)	0.3086 (3)	−0.48707 (15)	0.0497 (4)	
H8A	0.5789	0.2278	−0.5589	0.060*	
H8B	0.5298	0.3097	−0.4479	0.060*	
C9	0.7903 (3)	0.6028 (3)	−0.40700 (17)	0.0536 (5)	
H9A	0.6880	0.6021	−0.3681	0.064*	
H9B	0.8454	0.7225	−0.4244	0.064*	
C10	0.9345 (2)	0.5533 (2)	−0.32858 (15)	0.0453 (4)	
H10A	1.0405	0.5600	−0.3652	0.054*	
H10B	0.9784	0.6355	−0.2571	0.054*	
N1	0.74511 (16)	0.47391 (16)	0.01785 (11)	0.0332 (3)	
N2	0.21517 (18)	0.0548 (2)	−0.26913 (11)	0.0398 (3)	
N3	0.27441 (17)	0.01490 (17)	0.14458 (11)	0.0352 (3)	
N4	0.84911 (19)	0.36922 (19)	−0.30416 (11)	0.0373 (3)	
O1	0.81876 (17)	0.54823 (17)	−0.05681 (12)	0.0544 (4)	
O2	0.81128 (17)	0.52044 (18)	0.12078 (11)	0.0533 (3)	
O3	0.1114 (3)	0.1265 (3)	−0.31268 (14)	0.0847 (6)	
O4	0.2437 (3)	−0.0695 (3)	−0.31820 (14)	0.0838 (6)	
O5	0.14855 (16)	−0.13353 (15)	0.12339 (10)	0.0440 (3)	
O6	0.3436 (2)	0.0883 (2)	0.24304 (10)	0.0587 (4)	
O7	0.53339 (16)	0.33703 (18)	−0.21939 (10)	0.0463 (3)	
O8	0.08817 (14)	−0.11441 (14)	−0.09399 (10)	0.0371 (3)	
O9	0.7206 (2)	0.4823 (2)	−0.51108 (10)	0.0534 (3)	
H4A	0.762 (3)	0.371 (3)	−0.2657 (19)	0.053 (6)*	
H4B	0.931 (3)	0.333 (3)	−0.2646 (18)	0.047 (5)*	
H5	0.562 (3)	0.285 (3)	0.1470 (19)	0.048 (5)*	
H8	0.079 (4)	−0.160 (3)	−0.026 (2)	0.069 (7)*	

### Atomic displacement parameters (Å^2^)

**Table:**

	*U*^11^	*U*^22^	*U*^33^	*U*^12^	*U*^13^	*U*^23^
C1	0.0251 (6)	0.0320 (6)	0.0319 (7)	0.0093 (5)	0.0077 (5)	0.0079 (5)
C2	0.0269 (6)	0.0312 (6)	0.0266 (6)	0.0088 (5)	0.0033 (5)	0.0025 (5)
C3	0.0242 (6)	0.0244 (6)	0.0323 (7)	0.0082 (5)	0.0050 (5)	0.0033 (5)
C4	0.0273 (6)	0.0269 (6)	0.0288 (6)	0.0088 (5)	0.0066 (5)	0.0067 (5)
C5	0.0273 (6)	0.0283 (6)	0.0297 (7)	0.0090 (5)	0.0030 (5)	0.0033 (5)
C6	0.0221 (5)	0.0250 (6)	0.0352 (7)	0.0065 (5)	0.0045 (5)	0.0049 (5)
C7	0.0533 (9)	0.0447 (9)	0.0365 (8)	0.0164 (7)	0.0019 (7)	0.0028 (7)
C8	0.0473 (9)	0.0627 (11)	0.0332 (8)	0.0175 (8)	−0.0039 (7)	0.0003 (7)
C9	0.0680 (12)	0.0471 (10)	0.0506 (10)	0.0233 (9)	0.0134 (9)	0.0136 (8)
C10	0.0451 (9)	0.0448 (9)	0.0385 (8)	0.0042 (7)	0.0080 (7)	0.0061 (7)
N1	0.0245 (5)	0.0284 (6)	0.0452 (7)	0.0076 (4)	0.0049 (5)	0.0047 (5)
N2	0.0351 (6)	0.0491 (8)	0.0297 (6)	0.0079 (5)	0.0050 (5)	0.0014 (5)
N3	0.0343 (6)	0.0384 (6)	0.0337 (6)	0.0106 (5)	0.0084 (5)	0.0124 (5)
N4	0.0343 (6)	0.0486 (8)	0.0272 (6)	0.0108 (6)	0.0046 (5)	0.0101 (5)
O1	0.0419 (6)	0.0498 (7)	0.0578 (8)	−0.0079 (5)	0.0139 (6)	0.0147 (6)
O2	0.0385 (6)	0.0535 (7)	0.0484 (7)	−0.0044 (5)	−0.0036 (5)	−0.0010 (6)
O3	0.0871 (12)	0.1311 (16)	0.0459 (8)	0.0682 (12)	−0.0171 (8)	−0.0025 (9)
O4	0.1088 (14)	0.0908 (13)	0.0509 (9)	0.0491 (11)	−0.0020 (9)	−0.0255 (8)
O5	0.0432 (6)	0.0382 (6)	0.0473 (7)	0.0038 (5)	0.0130 (5)	0.0167 (5)
O6	0.0635 (8)	0.0691 (9)	0.0290 (6)	0.0013 (7)	0.0071 (5)	0.0107 (6)
O7	0.0364 (6)	0.0621 (8)	0.0379 (6)	0.0070 (5)	0.0117 (5)	0.0204 (5)
O8	0.0320 (5)	0.0316 (5)	0.0389 (6)	−0.0003 (4)	0.0047 (4)	0.0040 (4)
O9	0.0640 (8)	0.0711 (9)	0.0337 (6)	0.0321 (7)	0.0077 (5)	0.0185 (6)

### Geometric parameters (Å, °)

**Table:**

C1—O7	1.2426 (17)	C8—H8B	0.9700
C1—C2	1.4356 (19)	C9—O9	1.416 (2)
C1—C6	1.446 (2)	C9—C10	1.503 (3)
C2—C3	1.3665 (19)	C9—H9A	0.9700
C2—N2	1.4603 (19)	C9—H9B	0.9700
C3—O8	1.3378 (17)	C10—N4	1.482 (2)
C3—C4	1.4194 (19)	C10—H10A	0.9700
C4—C5	1.3817 (19)	C10—H10B	0.9700
C4—N3	1.4214 (17)	N1—O1	1.2203 (17)
C5—C6	1.3719 (19)	N1—O2	1.2233 (18)
C5—H5	0.91 (2)	N2—O3	1.197 (2)
C6—N1	1.4498 (18)	N2—O4	1.203 (2)
C7—N4	1.484 (2)	N3—O6	1.2184 (18)
C7—C8	1.502 (3)	N3—O5	1.2482 (18)
C7—H7A	0.9700	N4—H4A	0.87 (2)
C7—H7B	0.9700	N4—H4B	0.86 (2)
C8—O9	1.416 (3)	O8—H8	0.91 (3)
C8—H8A	0.9700		
			
O7—C1—C2	120.48 (13)	O9—C9—C10	111.16 (15)
O7—C1—C6	126.99 (13)	O9—C9—H9A	109.4
C2—C1—C6	112.53 (11)	C10—C9—H9A	109.4
C3—C2—C1	126.43 (12)	O9—C9—H9B	109.4
C3—C2—N2	118.38 (12)	C10—C9—H9B	109.4
C1—C2—N2	115.13 (12)	H9A—C9—H9B	108.0
O8—C3—C2	119.07 (12)	N4—C10—C9	108.79 (14)
O8—C3—C4	124.16 (12)	N4—C10—H10A	109.9
C2—C3—C4	116.77 (12)	C9—C10—H10A	109.9
C5—C4—C3	120.65 (12)	N4—C10—H10B	109.9
C5—C4—N3	118.42 (12)	C9—C10—H10B	109.9
C3—C4—N3	120.92 (12)	H10A—C10—H10B	108.3
C6—C5—C4	120.95 (13)	O1—N1—O2	122.51 (13)
C6—C5—H5	119.1 (13)	O1—N1—C6	119.61 (13)
C4—C5—H5	119.9 (13)	O2—N1—C6	117.88 (12)
C5—C6—C1	122.56 (12)	O3—N2—O4	123.56 (16)
C5—C6—N1	116.61 (13)	O3—N2—C2	118.88 (15)
C1—C6—N1	120.83 (12)	O4—N2—C2	117.55 (15)
N4—C7—C8	109.05 (15)	O6—N3—O5	121.80 (13)
N4—C7—H7A	109.9	O6—N3—C4	119.89 (13)
C8—C7—H7A	109.9	O5—N3—C4	118.30 (13)
N4—C7—H7B	109.9	C10—N4—C7	110.99 (13)
C8—C7—H7B	109.9	C10—N4—H4A	107.0 (14)
H7A—C7—H7B	108.3	C7—N4—H4A	109.7 (14)
O9—C8—C7	111.04 (15)	C10—N4—H4B	110.8 (14)
O9—C8—H8A	109.4	C7—N4—H4B	107.9 (14)
C7—C8—H8A	109.4	H4A—N4—H4B	110.4 (19)
O9—C8—H8B	109.4	C3—O8—H8	103.0 (16)
C7—C8—H8B	109.4	C8—O9—C9	109.86 (13)
H8A—C8—H8B	108.0		
			
O7—C1—C2—C3	177.37 (14)	C2—C1—C6—N1	178.50 (11)
C6—C1—C2—C3	−2.0 (2)	N4—C7—C8—O9	57.88 (19)
O7—C1—C2—N2	0.1 (2)	O9—C9—C10—N4	−58.2 (2)
C6—C1—C2—N2	−179.23 (12)	C5—C6—N1—O1	177.61 (13)
C1—C2—C3—O8	−176.85 (13)	C1—C6—N1—O1	−2.3 (2)
N2—C2—C3—O8	0.29 (19)	C5—C6—N1—O2	−3.30 (19)
C1—C2—C3—C4	3.7 (2)	C1—C6—N1—O2	176.81 (13)
N2—C2—C3—C4	−179.14 (12)	C3—C2—N2—O3	99.3 (2)
O8—C3—C4—C5	178.49 (12)	C1—C2—N2—O3	−83.3 (2)
C2—C3—C4—C5	−2.12 (19)	C3—C2—N2—O4	−80.3 (2)
O8—C3—C4—N3	−0.6 (2)	C1—C2—N2—O4	97.2 (2)
C2—C3—C4—N3	178.84 (12)	C5—C4—N3—O6	10.8 (2)
C3—C4—C5—C6	−1.0 (2)	C3—C4—N3—O6	−170.13 (14)
N3—C4—C5—C6	178.09 (12)	C5—C4—N3—O5	−168.37 (13)
C4—C5—C6—C1	2.8 (2)	C3—C4—N3—O5	10.7 (2)
C4—C5—C6—N1	−177.07 (12)	C9—C10—N4—C7	54.78 (19)
O7—C1—C6—C5	179.29 (14)	C8—C7—N4—C10	−54.73 (19)
C2—C1—C6—C5	−1.38 (19)	C7—C8—O9—C9	−61.7 (2)
O7—C1—C6—N1	−0.8 (2)	C10—C9—O9—C8	62.0 (2)

### Hydrogen-bond geometry (Å, °)

**Table:**

*D*—H···*A*	*D*—H	H···*A*	*D*···*A*	*D*—H···*A*
O8—H8···O5	0.91 (3)	1.75 (3)	2.571 (2)	149 (2)
N4—H4A···O7	0.87 (2)	1.87 (2)	2.715 (2)	164 (2)
N4—H4B···O2^i^	0.86 (2)	2.31 (2)	2.962 (2)	132.4 (17)
N4—H4B···O5^ii^	0.86 (2)	2.42 (2)	2.967 (2)	121.5 (17)
N4—H4B···O3^iii^	0.86 (2)	2.54 (2)	3.206 (3)	134.3 (17)

Symmetry codes: (i) −*x*+2, −*y*+1, −*z*; (ii) −*x*+1, −*y*, −*z*; (iii) *x*+1, *y*, *z*.
